# Gender neutral HPV vaccination programs: Reconsidering policies to expand cancer prevention globally

**DOI:** 10.3389/fpubh.2023.1067299

**Published:** 2023-02-21

**Authors:** J. Andrew Dykens, Caryn E. Peterson, Hunter K. Holt, Diane M. Harper

**Affiliations:** ^1^Department of Family and Community Medicine, College of Medicine, University of Illinois Chicago, Chicago, IL, United States; ^2^Center for Global Health, College of Medicine, University of Illinois Chicago, Chicago, IL, United States; ^3^Cancer Center, College of Medicine, University of Illinois Chicago, Chicago, IL, United States; ^4^Division of Epidemiology and Biostatistics, School of Public Health, University of Illinois Chicago, Chicago, IL, United States; ^5^Department of Family Medicine, School of Medicine, University of Michigan, Ann Arbor, MI, United States; ^6^Department of Obstetrics & Gynecology, School of Medicine, University of Michigan, Ann Arbor, MI, United States; ^7^Department of Women's Studies, College of Literature, Science and the Arts, University of Michigan, Ann Arbor, MI, United States

**Keywords:** HPV, cervical cancer, cancer prevention, policy, vaccine program

## Abstract

Human papillomavirus (HPV) infection is responsible for many cancers in both women and men. Cervical cancer, caused by HPV, is the fourth most common cancer among women worldwide, even though it is one of the most preventable cancers. Prevention efforts include HPV vaccination, however these programs remain nascent in many countries. In 2020 the World Health Assembly adopted the Global Strategy for cervical cancer elimination including a goal to fully vaccinate 90% of girls with the HPV vaccine by the age of 15. However, very few countries have reached even 70% coverage. Increased vaccine availability in the future may allow the opportunity to vaccinate more people. This could add to the feasibility of introducing gender-neutral HPV vaccination programs. Adopting a gender-neutral HPV vaccine approach will reduce HPV infections transmitted among the population, combat misinformation, minimize vaccine-related stigma, and promote gender equity. We propose approaching programmatic research through a gender-neutral lens to reduce HPV infections and cancers and promote gender equality. In order to design more effective policies and programs, a better understanding of the perspectives of clients, clinicians, community leaders, and policy-makers is needed. A clear, multi-level understanding of these stakeholders' views will facilitate the development of target policy and programs aimed at addressing common barriers and optimizing uptake. Given the benefit of developing gender-neutral HPV vaccination programs to eliminate cervical cancer and address other HPV associated cancers, we must build knowledge through implementation research around this topic to inform policy-makers and funders for future policy shifts.

## Introduction

Human papillomavirus (HPV) affects both women and men and is the most common sexually transmitted infection in the world (1). There are more than 100 HPV types of which 13 are classified as oncogenic. HPV is highly prevalent globally, with an estimated 80%−90% of women and men acquiring infection in their lifetime (2, 3). Oncogenic HPV types are responsible for anogenital and cutaneous warts and several cancers including oropharyngeal, anogenital, cervical, anal, vulvar, vaginal, and penile (1).

Cervical cancer is the predominant HPV-related cancer and is the fourth most common cancer among women worldwide, despite effective prevention strategies (4). Global estimates show that cervical cancer occurred in 604,127 new cases and led to 341,831 deaths in 2020 (5). It is the most common type of cancer-related mortality among women in 42 countries, the majority living in Sub-Saharan Africa (4).

While cervical cancer incidence rates are declining or stagnating in high-income countries, the absolute number of cases continue to rise in low- and lower-middle-income countries (LMIC) (4). Models show that cervical cancer burden will increase by 17% to 708,000 cases and mortality will increase by 42% to 442,926 deaths in the year 2030 (6, 7). The most significant rise will be in LMICs, where 84% of incident cervical cancer cases and 90% of cervical cancer deaths occur (4).

Virtually all cases of invasive cervical cancer are caused by infection with high-risk (oncogenic) HPV infection types (8). Most infections (90%) are cleared through a normal immune-response within 1 year, with ~10% of all infections with an oncogenic HPV type progressing to a precancer or invasive cervical cancer (9). HPV 16 and 18 are responsible for about 70% of cervical cancer worldwide (10) while >90% of cases worldwide are caused by HPV types 16, 18, 31, 33, 45, 52, and 58 (11).

The higher rates of cervical cancer incidence and mortality in LMICs is attributable to the relative lack of high-quality cervical cancer screening and lack of widespread high-quality treatment of preinvasive/invasive cervical cancer in LMICs rather than significant differences in HPV infection rates (12). Higher rates, however, do not appear to be broadly associated with differences in cervical infection with oncogenic HPV types (10) (though there are higher rates of HPV among women infected with human immunodeficiency virus).

HPV vaccination programs remain nascent in several countries, particularly LMICs. Globally, there are 122 World Health Organization (WHO) countries and territory member states, and 27 non-members with HPV vaccination on the national routine immunization schedule. As of 2022, eight of 29 (28%) low-income countries and 22 of 51 (43%) lower-middle income countries had introduced HPV vaccine programs (13). Meanwhile, 72% of low-income countries and 57% of lower-middle income countries are yet to include HPV vaccination into their national immunization programs (14). Cost is a significant factor in a country's ability to initiate and maintain a program (15). The actual cost of the vaccine, which increases over time, initially accounts for 51% of total program costs (16). Other direct medical costs which tend to decrease over time include cold chain, workforce education, monitoring and evaluation, community education, and vaccination campaigns (14, 17).

### Current global HPV vaccination program guidance and progress

In 2020 the World Health Assembly adopted the Global Strategy for the Elimination of Cervical Cancer. This strategy includes targets for HPV vaccination, and cervical cancer screening and treatment. Specifically, that 90% of girls will be fully vaccinated by the age of 15; that 70% of women will be screened using a high-performance test by the age of 35, and again by the age of 45; and that 90% of women with pre-cancer or invasive cancer will be treated or managed. Achieving these targets by 2030 will ensure that all countries reach and maintain a cervical cancer incidence rate of below four per 100,000 women (10).

Many transmission-dynamic models developed by the WHO Cervical Cancer Elimination Modeling Consortium show that 70% coverage through a girls-only HPV vaccination approach at 15 years of age leads to disease reduction (18). However, very few countries have reached 70% coverage. LMIC HPV vaccination coverage is 16% (8%−31% among 51 countries) for the first dose and 12% (5%−24% in 43 countries) for the second dose. Likewise, in Gavi eligible countries in 2018 (14 countries), 12% (4%−32%) of the population received one dose, and 7% (1%−21%) received the second dose in six countries (19). Based on these deficient coverage levels, neither disease reduction nor herd immunity will occur. In order to bolster the beneficial effects of the HPV vaccine, we must consider a gender-neutral approach. Recent models have shown that vaccinating boys in an environment of female coverage below 50% may be cost-effective, depending upon the prevalence of HPV-related disease and available resources (20). In addition, a systematic review that primarily included studies from high income countries showed that as long as vaccine price remained low, a gender neutral vaccination program was cost-effective if coverage was low. The same review did reinforce earlier findings that gender-neutral vaccination was less cost-effective than when targeting only girls aged 9–18 years if coverage for females was above 75% (21), however achieving this level of coverage in some LMICs is challenging.

The WHO has recently expanded recommended ages among females and added males within a secondary target group for HPV vaccination (22). Of the 141 global HPV vaccination programs, there are 43 countries and 4 territories that have gender-neutral HPV vaccine schedules (23). All of these programs are in high income countries and upper middle-income countries (19) except for a single program. Bhutan became the first LMIC and the first country in South East Asia to adopt gender-neutral vaccination policy in September 2020 (24).

Female-only HPV vaccination programs have several shortcomings (25) including the consideration of only cervical cancer as a HPV transmission outcome (21) and multiple additional assumptions: (1) of monogamy or serial monogamy with few lifetime partners without the consideration of polygamous societies (20); (2) of heterosexual relationships, thus discounting the potential for HPV spread through bisexual contact and by way of men who have sex with men (26); (3) of penile-vaginal intercourse, thus minimizing the consideration of digital and oral spread of HPV; (4) of the presence of gender equity without consideration of women's structural barriers (such as lack of autonomy, early marriages, and lack of education); and (5) of a uniform geographic and other social determinant acceptability of HPV vaccination. These assumptions may contribute to inequities as well as mistrust and misinformation.

### One dose HPV vaccination approach

There is mounting evidence that a one-dose approach may prove effective (27–32). Currently, ongoing studies (33) through the Costa Rica Vaccine Trial (31), a multicenter cohort study in India (32), and the industry-sponsored PATRICIA trial (28) will inform future guidelines. Recently, The WHO Strategic Advisory Group of Experts on Immunization (SAGE) recommended updating the dosing schedule to one or two doses for girls aged 9–14 and women 15–20 years and concluded that a single dose is comparable to a 2-dose schedule (34). Compared with a two-dose HPV vaccination schedule, one-dose HPV vaccination could reduce many barriers. For instance, studies highlight program costs, ease of administration, multi-cohort vaccination delivery, and increased HPV vaccine program adoption in populations with limited access to healthcare and a high burden of cervical cancer (33).

With growing evidence of the potential efficacy of a one-dose approach, new predictive modeling is underway. Additional simulation models suggest that one-dose vaccination has similar health benefits to a two-dose regimen but also simplifies vaccine delivery, reduces costs, and helps to alleviate vaccine supply constraints (35, 36). Countries that have yet to implement an HPV vaccination program or have low coverage but a high burden of HPV-related diseases may benefit by implementing one-dose vaccination (37). Available resources in LMICs favor a one-dose approach, but data are needed to support adoption (38). Evidence may be sufficient to alter existing guidelines by 2025 (39).

### Considering gender-neutral vaccination programs

Eliminating cervical cancer means reducing the incidence to <4/100,000. The current approach, with sustained vaccination for girls only, will change disease rates in 70 years if 100% coverage occurs (40–43). If it is impossible to reach 70% coverage for girls, superior cancer control will need gender-neutral immunization in order to more rapidly impact disease rates (44). Recent modeling assumes HPV vaccine efficacy is >85% and confers lifelong protection. Extending the target-age range of girls and women and focusing on boys is more cost-effective than giving a second dose to girls aged 9–14 when the outcomes are the maximum ICER (incremental cost-effectiveness ratio) and the minimum NNT (number needed to treat). This result applies to India, Vietnam, Uganda, and Nigeria, the countries under study (33).

The epidemiological and economic considerations about vaccinating boys should focus on the benefits in terms of disease reduction, the feasibility and incremental marginal costs of increasing vaccination coverage among girls vs. introducing a gender-neutral program (41, 45). Importantly, gender-neutral HPV vaccination programs will also depend on population acceptability (14, 36, 46, 47) and political viability (47–50).

## Discussion

### Forward perspective

It is time to reconsider a gendered approach to vaccination to best respond to the WHO's call to eliminate cervical cancer. To date, modeling has primarily been driven by reconciling constraints (vaccine availability and financial barriers) with consideration of cost-effectiveness. With advancing knowledge that potentially reinforces a single-dose approach, the potential to vaccinate more people with current vaccine supplies emerges (i.e., the transition to single dose looks to be promising and is a reasonable expectation to be considered). Other possibilities that may alter vaccine availability include: (1) vaccine cost decline, (2) increased production, and (3) changes in licensing policy. With these considerations, a shift in resource availability may result in opportunities to transition global HPV vaccine programs to alternative approaches. There is a need to continue reworking HPV vaccination models and inform potential approaches through implementation research. Currently, research on implementing a gender-neutral HPV vaccination approach is lacking, and understanding the context in which gender-neutral programs will be successfully adopted is critical.

A gender-neutral HPV vaccination approach will advance the health of both male and female populations. In women, HPV vaccination must include at least one primary HPV screening test in the woman's mid-life. HPV vaccines are incomplete in their genotype prevention, and screening is necessary to reduce the incidence to achieve the WHO elimination goal of <4/100,000. The screening services are likely to be more effective in a population already vaccinated as the underlying prevalence of HPV infection will be lower. Likewise, the treatment services will go to fewer numbers of women who still develop CIN 3 or early cervical cancers.

### Knowledge gaps informing a gender neutral approach in LMICs

Adopting a gender-neutral HPV vaccine approach will reduce HPV infections transmitted among the population. We must plan for HPV vaccine uptake, combat misinformation, minimize vaccine-related stigma, and promote gender equity (46). HPV is not a virus that only infects female epithelium; instead, it is a gender-neutral infection. We propose approaching programmatic research through a gender-neutral lens to reduce HPV infections and cancers and promote gender equality (51).

To achieve these goals, we must begin to understand the local community-based customs that will affect the acceptability of gender-neutral vaccination programs in LMICs. Having male and female population endorsement is critical to a gender-neutral approach to HPV vaccination. Once a community-based agreement pushes for gender-neutral vaccination, we must have the data to show the effectiveness of a single HPV vaccine dose in males, as we currently do for females. Designing and supporting variable dose HPV vaccine studies in males is critical for long-term follow-up. There is little literature monitoring male serologic titers and HPV-related outcomes (52).

In addition, implementation research is needed to inform the acceptability, adoption, appropriateness, feasibility, fidelity, implementation cost, penetration, and sustainability of transitioning to a gender neutral approach. There is some recent literature describing the barriers toward HPV vaccinations for boys. However these reports are mostly in high-income countries or upper middle-income countries (53). We are not aware of literature on this topic in the LMIC context. Given the benefit of developing gender neutral HPV vaccination programs in the effort to eliminate cervical cancer, it is imperative that we build knowledge around this topic in order to inform policy-makers and funders in consideration of the possibility for future policy shifts.

### Limitations

There are some limitations to the stated argument in favor of advancing research in order to inform the consideration of developing gender neutral HPV vaccination programs. Foremost, there is a noted distinction between the WHO goal of elimination of cervical cancer and individual country-level health ministry considerations regarding research evaluating cost effectiveness of HPV vaccination strategies. It is not always possible to compare all variables across these perspectives. Therefore, these goals may not align in many cases and contextual considerations may weigh certain variables in lieu of others. Secondly, given that resources are currently limited and arguments relative to future availability of the HPV vaccine are, ultimately, conjecture, the current modeling limitations create gaps in our ability to precisely estimate the effects of any potential change in resources. Thirdly, our call to action does not fully consider currently unknown, or unconsidered, repercussions of a significant change in vaccination strategy such as effects on supply chains and distribution channels of the HPV vaccine between and within countries. Our hope is that implementation research would help to shine light on these types of challenges, thus informing future policy-makers at all levels. In addition, it is noted that there is a lack of effective HPV screening methods in men and research exploring male HPV infection is deficient (54). Additional studies assessing the cost-effectiveness of HPV testing in men and modeling studies to assess the effects in females of adding males to existing HPV vaccination programs are needed (55). Lastly, a major unaccounted shift in vaccine technology could also significantly alter these considerations. Ultimately, these limitations may also be concurrently viewed as additional evidence of the need for more research.

## Conclusion

HPV and HPV vaccination are sensitive topics and are commonly associated with misinformation, rumors, and stigma (56–58). In order to design more effective policies and programs, a better understanding of the perspectives of clients, clinicians, community leaders, and policy-makers is needed. A clear, multi-level understanding of these stakeholders' views will facilitate the development of target policy and programs aimed at addressing common barriers and optimizing uptake. Given the benefit of developing gender-neutral HPV vaccination programs to eliminate cervical cancer, we must build knowledge around this topic to inform policy-makers and funders for future policy shifts.

## Author contributions

JD, CP, HH, and DH contributed to the conceptualization, analysis, interpretation and writing of the paper. All authors contributed to the article and approved the submitted version.
